# Imaging of cerebrospinal fluid leakage from the cribriform plate

**DOI:** 10.1016/j.radcr.2025.01.004

**Published:** 2025-01-20

**Authors:** Tomoki Sato, Yohei Morishita, Jun Suzuki, Ryu Narita, Keisei Uehara, Erina Kato, Kei Takase

**Affiliations:** a Department of Diagnostic Radiology, Tohoku University Graduate School of Medicine, 1-1 Seiryo-machi, Aoba-ku, Sendai, Miyagi 980-8574, Japan; b Department of Otolaryngology, Head and Neck Surgery, Tohoku University Graduate School of Medicine, 1-1 Seiryo-machi, Aoba-ku, Sendai, Miyagi 980-8574, Japan; c Department of Diagnostic Radiology, Takeda General Hospital, 3-27 Yamaga-machi, Aizuwakamatsu, Fukushima 965-8585, Japan

**Keywords:** Cerebrospinal fluid leakage, Cribriform plate, Olfactory fissure, Meningocele, Fluid accumulation in the sphenoid sinus

## Abstract

Cerebrospinal fluid leakage from the skull base occurs when the subarachnoid space communicates with the extracranial space. Cerebrospinal fluid leakage from the cribriform plate is often difficult to diagnose because the fistula is small. If the fistula can be identified before surgery, this can contribute greatly to treatment. We present 2 cases of cerebrospinal fluid leakage from the cribriform plate with tiny fistulas, which we were able to diagnose before surgery with CT and MRI.

## Introduction

Cerebrospinal fluid (CSF) leakage from the skull base occurs when the subarachnoid space communicates with the extracranial space. Open communication of the subarachnoid space and CSF leakage may cause serious infections, such as meningitis. If the CSF leakage does not improve with conservative treatment, the fistula must be closed surgically [1].

Imaging is used to identify fistulas and signs of intracranial infections. In cases of CSF leakage from the cribriform plate, the fistula is often small, and bone defects may not be identifiable using computed tomography (CT) [2].

We present 2 cases of CSF leakage from the cribriform plate with tiny fistulas.

## Case report

### Case 1

The patient was a woman in her 50 s, with a history of hypertension, dyslipidemia, and sigmoid colon cancer. She noticed rhinorrhea from her left nostril. One month earlier, she had visited a local doctor because she had a headache and fever, and she was treated with antibiotics. However, the rhinorrhea persisted, and a CSF leak was suspected. A lumbar puncture was performed, and there were no findings suggestive of meningitis.

**Fig. 1A:**
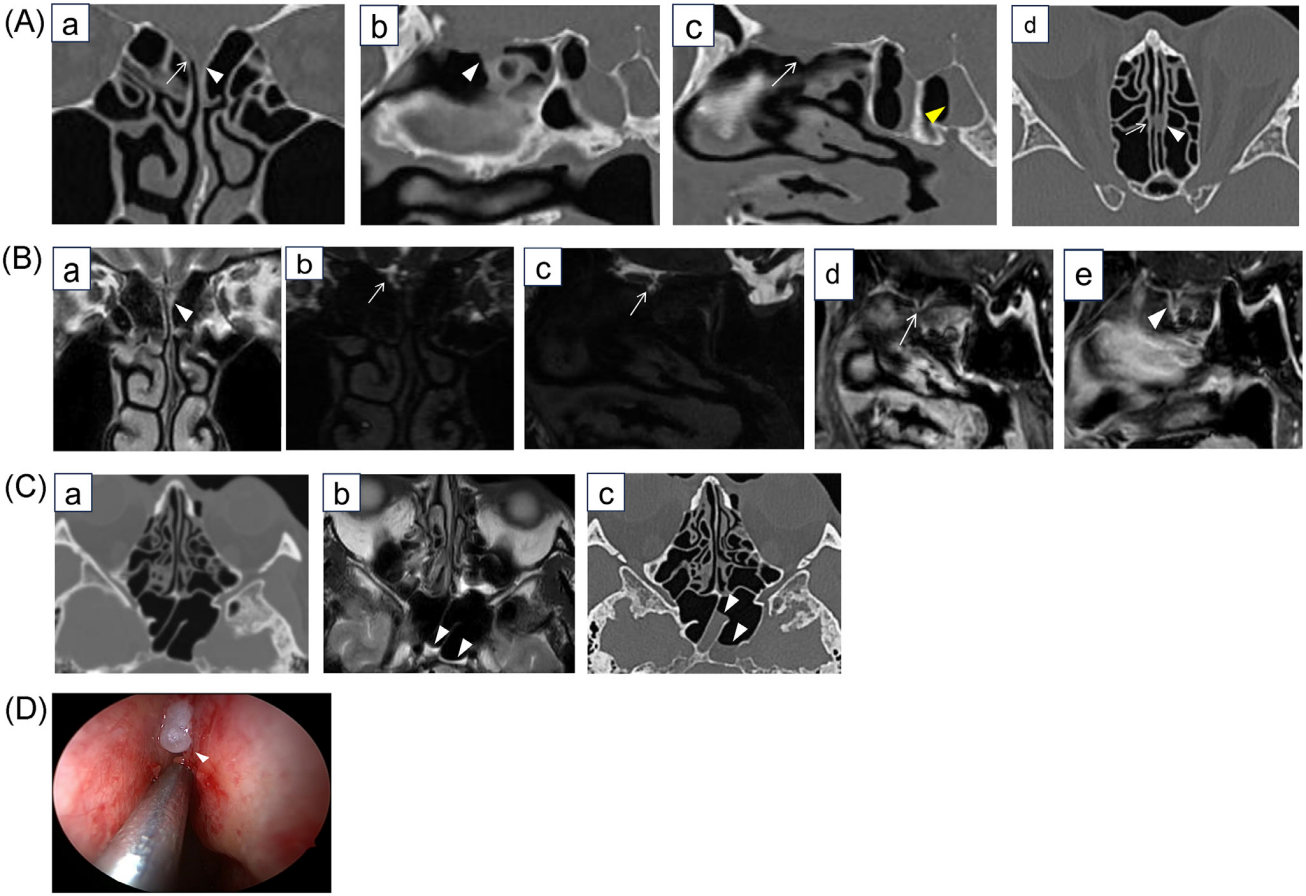
**CT (**a) Oblique coronal plane aligned perpendicular to the base of the skull. (b) Oblique sagittal plane aligned with the left olfactory fissure. (c) Oblique sagittal plane aligned with the right olfactory fissure. (d) Oblique axial plane aligned parallel to the base of the skull. Bilateral cribriform plates were indistinct, but it was unclear whether there were bone defects (a-c). A soft tissue density protruded from the cribriform plate in both olfactory fissures with left-sided predominance, which may have indicated the site of leakage (a-d). CSF leakage from the left side was suspected on the basis of the patient's symptoms and an air-fluid level in the left sphenoid sinus (c) (yellow arrowhead). **1B. MRI**. (a) Coronal T2WI. (b) Coronal 3D-MR cisternography. (c) 3D-MR cisternography oblique sagittal plane aligned with the right olfactory fissure. (d) 3D-MR cisternography oblique sagittal plane aligned with the right olfactory fissure. (e) Gd-T1WI oblique sagittal plane aligned with the left olfactory fissure. Coronal T2WI showed a high signal intensity in the left olfactory fissure, which corresponded to the soft tissue density seen on CT (a) (arrowhead). On 3D-MR cisternography, the signal in the left olfactory fissure was unclear, and there was a high signal intensity continuous from the subarachnoid space to the right olfactory fissure (b and c) (arrows). Gd-TIWI showed a meningocele-like structure protruding from the cribriform plate on both sides (d and e) (arrow and arrowhead). **1C.** (a) Axial CT image. (b) Axial T2WI MRI 3 hours after the CT images were obtained. (c) Axial CT image 4 days after the MRI images were obtained. (d) The air-fluid level in the left sphenoid sinus increased in size between the CT and MRI imaging, suggesting persistent leakage of CSF in the left cribriform plate (a-c) (arrowheads in b and c indicate the accumulating fluid collection). No air-fluid level was observed in the right sinus. **1D. Endoscopy** A meningocele (arrowhead) measuring 1-2 mm in size was found in the left olfactory fissure during endoscopic surgery. CT, computed tomography; CSF, cerebrospinal fluid; MRI, magnetic resonance imaging; T2WI, T2-weighted imaging.

Head CT and magnetic resonance imaging (MRI) were performed to identify the site of the CSF leak. CT revealed that bilateral cribriform plates were unclear, and a soft tissue density protruded from the cribriform plate in both olfactory fissures, with left-sided predominance. There was an air-fluid level in the left sphenoid sinus; therefore, left-sided CSF leakage was suspected. MRI showed hyperintensity on coronal T2-weighted images (T2WI) in the left olfactory fissure, and a fluid component was suspected. Three-dimensional (3D) MR cisternography showed a high signal intensity in the right olfactory fissure, continuous with the subarachnoid space, and an unclear fluid signal in the left olfactory fissure. Gadolinium-enhanced T1-weighted images (Gd-T1WI) showed meningoceles protruding from the cribriform plate in both olfactory fissures. MRI was performed approximately 3 h after CT, and the amount of fluid in the left sphenoid sinus increased over this short period of time, a finding that was thought to reflect persistent leakage (Fig. 1A).

Endoscopic CSF leak repair was performed, which revealed a meningocele measuring 1-2 mm in the left olfactory fissure and CSF leakage from the left cribriform plate. The left nasal discharge symptoms disappeared after surgery.

### Case 2

**Fig. 2A:**
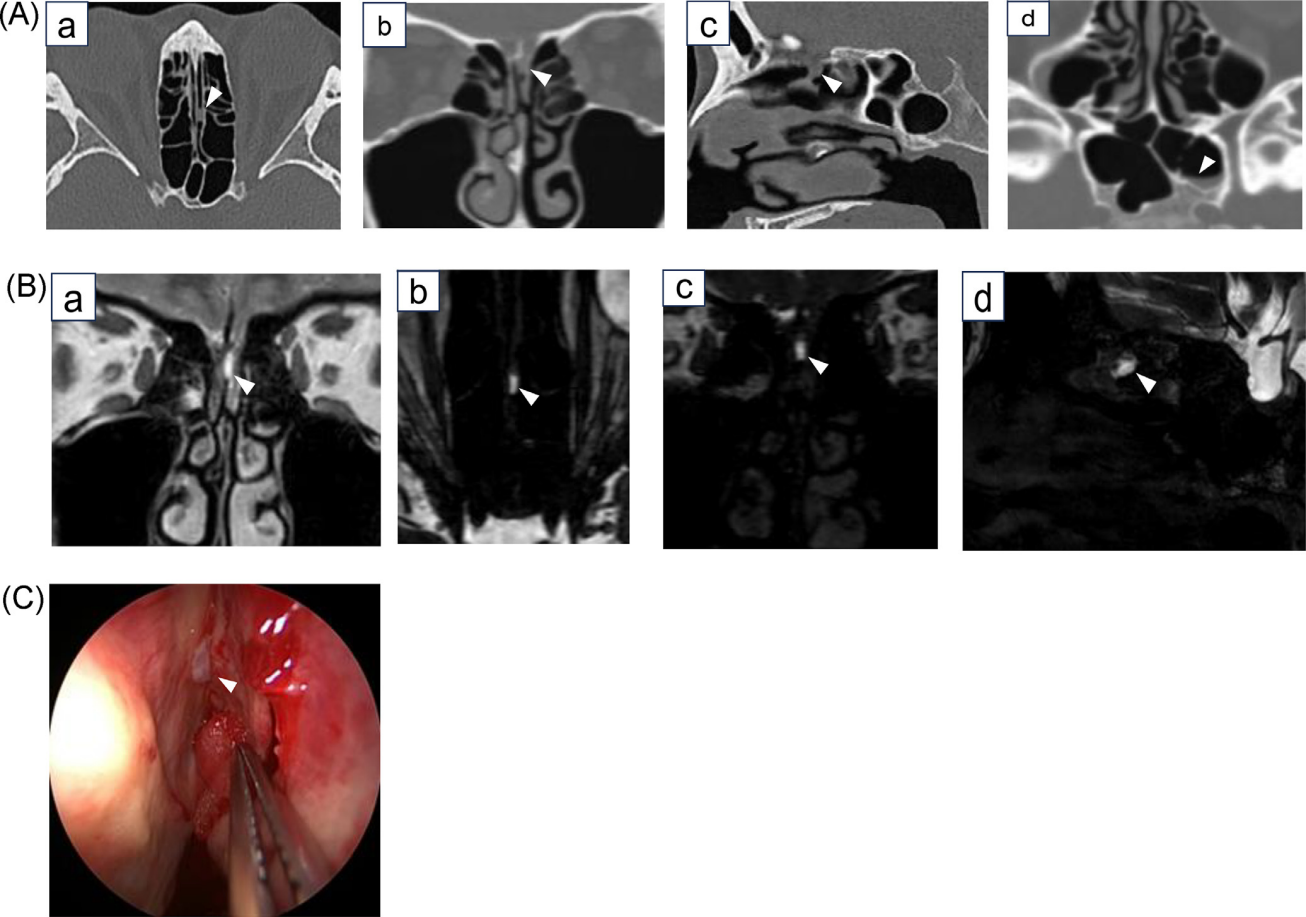
**CT**. (a) ) Oblique axial plane aligned parallel to the base of the skull. (b) Oblique coronal plane aligned perpendicular to the base of the skull. (c) Oblique sagittal plane aligned with the left olfactory fissure. (d) Axial plane. The left cribriform plate was unclear, and there was a soft tissue density continuous with the subarachnoid space (a-c) (arrowheads). An accumulation in the left sphenoid sinus was thought to be leaked CSF (d) (arrowhead). 2B. MRI. (a) Coronal T2WI. (b) 3D-MR cisternography oblique axial plane aligned parallel to the base of the skull. (c) 3D-MR cisternography oblique coronal plane aligned perpendicular to the base of the skull. (d) 3D-MR cisternography oblique sagittal plane aligned with the left olfactory fissure. T2WI and 3D-MR cisternography showed high signal intensity in the left olfactory fissure, suggesting fluid (a-d) (arrowheads). However, communication with the subarachnoid space was not evident. 2C. **Endoscopy**. A meningocele (arrowhead) between the left superior nasal turbinate and the nasal septum was observed. The fistula measured approximately 1-2 mm in size. CT, computed tomography; CSF, cerebrospinal fluid; MRI, magnetic resonance imaging; T2WI, T2-weighted imaging; 3D, three-dimensional.

The patient was a woman in her 30 s with a history of systemic lupus erythematosus, cerebral venous sinus thrombosis, and intracranial hypertension. She had persistent nasal discharge, and CSF leakage had been suspected for 4 years. Because she did not request further investigation, she had not received treatment. Head CT and MRI were performed subsequently, when the patient requested detailed examination and treatment. Head CT revealed a soft tissue density in the left olfactory fissure continuous with the left cribriform plate, and there was an air-fluid level in the left sphenoid sinus. Head MRI showed high signal intensity in the left olfactory fissure on coronal T2WI, suggesting a fluid component. 3D-MR cisternography also showed a fluid signal in the left olfactory fissure, but its continuity with the arachnoid space was unclear (Fig. 2A).

Intraoperatively (endoscopic CSF leak repair), a meningocele was found between the left superior nasal turbinate and the nasal septum, and CSF leakage was observed. The fistula in the olfactory fissure measured approximately 1-2 mm in size. After CSF leak closure, nasal discharge disappeared; however, symptoms of increased intracranial pressure recurred, and lumboperitoneal shunt surgery was performed 1 month later.

## Discussion

Imaging modalities for detailed examination of CSF leakage comprise high-resolution CT (HRCT), MRI, CT cisternography, Gd-enhanced MR-cisternography, and radionuclide cisternography. CSF leakage requires the presence of bone defects and meningeal defects, and HRCT mainly evaluates bone defects [[3], [4], [5]].

According to a recent meta-analysis, the sensitivity of HRCT for nasal discharge caused by CSF leakage is 75.5 %, and the specificity is approximately 85 % [4]. Additionally, HRCT has the advantage of being non-invasive and relatively inexpensive, and is quickly and easily accessible compared with MRI [4,6]. It is easy to identify a large bone defect with HRCT, but it is difficult to find bone defects that measure < 2 mm [4,7,8]. However, in cases of CSF leakage from the cribriform plate, the bone defect may be small, and the only finding may be a local soft tissue density in the olfactory fissure, or a meningocele [2]. Additionally, it is not uncommon to see thinning or separation of bones at the base of the skull [9]. Furthermore, it is difficult to distinguish between bone defects indicative of active leakage from thinning or separation of bones without pathological significance [[3], [4], [5],^7^].

MRI is useful for evaluating the content of soft tissue density lesions seen on CT [5]. In other words, if it can be confirmed that the CSF intensity continues outside the skull on T2WI, there is a possibility of CSF leakage. It is also important to evaluate whether a meningocele is accompanied by brain herniation [5,10,11]. In addition to conventional 2-dimensional T2WI, there are also reports that 3D-MR cisternography is useful to depict the continuity of the CSF intensity from the subarachnoid space to the extracranial space [3]. However, 3D-MR cisternography can cause false-positive or false-negative results due to magnetic susceptibility artifacts; therefore, this imaging technique should be used as a complement to conventional 2-dimensional T2WI [11]. The reported sensitivity of MRI is 89 %, which can be increased to 96 % when combined with CT because these imaging techniques can complement each other [12].

An air-fluid level in the sinuses near the fistula can be seen with both CT and MRI, which can indirectly suggest the presence of CSF leakage. However, it should be noted that the sinus area often has fluid accumulation due to inflammatory changes [3,5,13].

CT cisternography, intrathecal Gd-enhanced MR cisternography, and radionuclide cisternography are invasive tests in which contrast agents and radionuclides are administered into the subarachnoid space. With CT cisternography and intrathecal Gd-enhanced MR cisternography, it is possible to identify fistulas where contrast medium leaks from the subarachnoid space. The reported sensitivity of CT cisternography is 85 %-92 % for active leaks and 40 % for inactive leaks [3,5,12,14,15]. The reported sensitivity of Gd-enhanced MR cisternography is 87 %-100 % for active leaks and 70 % for inactive leaks [3,11,16,17], and some studies report that Gd-enhanced MR cisternography is superior to CT cisternography [11,13]. However, although the safety of administering Gd contrast agents into the subarachnoid space has been suggested, this procedure has not been approved by the US Food and Drug Administration [3,7,11,13]. Although radionuclide cisternography can be used to assess the presence or absence of leakage, it is difficult to identify fistulas with this method owing to resolution issues [1,3]. The reported sensitivity of radionuclide cisternography is 28 %-76 % [15,18].

In Case 1, tiny bone defects were suspected in bilateral cribriform plates, and there were soft tissue densities in both olfactory fissures on CT. Although 3D-MR cisternography showed continuous CSF intensity from the subarachnoid space to the right olfactory fissure, the CSF intensity was unclear on the left side. The indistinct CSF signals on 3D-MR cisternography were thought to be due to magnetic susceptibility artifacts because coronal T2WI revealed high signals in the left olfactory fissure. Because the fluid accumulation in the sphenoid sinus was localized to the left side, we were able to diagnose CSF leakage from the left olfactory fissure. Furthermore, Gd-T1WI performed to evaluate intracranial infections showed the meninges falling through the fistula, which may be a direct finding associated with a fistula [5]. In this case, although abnormalities in bilateral cribriform plates were suspected on imaging, the presence of the clinical symptom of left-sided nasal discharge and the indirect finding of fluid accumulation in the left sphenoid sinus enabled the diagnosis of active CSF leakage from the left cribriform plate.

In Case 2, although the fistula was tiny, we suspected a fistula because a bone defect and soft tissue density attached to the left cribriform plate were visible with CT. We suspected the presence of the fistula because the soft tissue density in the left olfactory fissure on CT showed CSF signals on MRI although 3D-MR cisternography could not visualize continuity between the CSF signals and the subarachnoid space. We also identified an air-fluid level in the sphenoid sinus on the affected side and changes in the amount of liquid in a relatively short period, which we consider an indirect finding suggesting active CSF leakage. We detected fluid accumulation in the sphenoid sinus on the affected side, which indirectly supported CSF leakage.

In conclusion, fistulas leading to CSF leakage from the cribriform plate may be small, making diagnosis difficult. Accurate radiological diagnosis can be made by combining multiple findings on CT and MRI. Additionally, informing the surgeon of the fistula site before surgery can contribute to treatment.

### Ethics statement

This study was approved by the institutional review board of our hospital.

## Patient consent

Written informed consents were obtained from the patients for publication of their anonymized information in this article.
